# Repply to: When technology advances faster than competence: the risk of deskilling in obstetrics and gynecology

**DOI:** 10.61622/rbgo/2026rbgo108L22R

**Published:** 2026-10-13

**Authors:** Agnaldo Lopes da Silva, Eduardo Cordioli, Sérgio Podgaec, Marcos Felipe Silva de Sá, Maria Celeste Osorio Wender

**Affiliations:** 1 Universidade Federal de Minas Gerais Belo Horizonte MG Brazil Universidade Federal de Minas Gerais, Belo Horizonte, MG, Brazil; 2 Hospital e Maternidade Santa Joana São Paulo SP Brazil Hospital e Maternidade Santa Joana, São Paulo, SP, Brazil; 3 Sociedade Beneficente Israelita Brasileira Albert Einstein São Paulo SP Brazil Sociedade Beneficente Israelita Brasileira Albert Einstein, São Paulo, SP, Brazil; 4 Universidade de São Paulo Faculdade de Medicina São Paulo SP Brazil Faculdade de Medicina, Universidade de São Paulo, São Paulo, SP, Brazil; 5 Universidade de São Paulo Faculdade de Medicina Ribeirão Preto SP Brazil Faculdade de Medicina, Universidade de São Paulo, Ribeirão Preto, SP, Brazil; 6 Universidade Federal do Rio Grande do Sul Porto Alegre RS Brazil Universidade Federal do Rio Grande do Sul, Porto Alegre, RS, Brazil

Dear Editor,

We thank the author for the thoughtful comments on our article about the risk of deskilling associated with the increasing use of artificial intelligence in obstetrics and gynecology. We agree that artificial intelligence will become part of routine medical practice. We did not intend to argue against its use, but to emphasize that technology should support clinical practice without replacing the knowledge, judgment, and technical abilities required of physicians.

The examples of obstetric emergencies are particularly relevant. Situations such as persistent fetal bradycardia, recurrent late decelerations, and severe hemorrhage require immediate recognition and action, even when artificial intelligence is unavailable or its recommendations are inappropriate. Physicians must therefore retain the ability to assess these situations and initiate treatment independently.

Artificial intelligence may also be valuable in medical education, especially through simulation and case discussions. However, its use should strengthen clinical reasoning rather than encourage dependence on automated recommendations. Training programs should continue to assess essential clinical and procedural skills and should prepare physicians to recognize the limitations of these systems.

The comparison with the development of imaging in cervical cancer staging is also helpful. New technologies have improved diagnosis while complementing clinical examination. Artificial intelligence may follow a similar path, although its recommendations can sometimes be less transparent and may create a false sense of confidence.

We agree that technological progress and the preservation of fundamental clinical skills should not be viewed as opposing goals. The best approach is to ensure that physicians are able to use artificial intelligence appropriately while maintaining the competence needed to care for patients when technology is unavailable, inadequate, or misleading. We appreciate the opportunity to clarify our position and thank the author for emphasizing the importance of responsibly integrating artificial intelligence into obstetric and gynecologic practice.

